# Using Intervention Mapping to Develop a Workplace Digital Health Intervention for Preconception, Pregnant, and Postpartum Women: The Health in Planning, Pregnancy and Postpartum (HiPPP) Portal

**DOI:** 10.3390/ijerph192215078

**Published:** 2022-11-16

**Authors:** Claire Blewitt, Melissa Savaglio, Seonad K. Madden, Donna Meechan, Amanda O’Connor, Helen Skouteris, Briony Hill

**Affiliations:** 1 Health and Social Care Unit, School of Public Health and Preventive Medicine, Monash University, Melbourne, VIC 3004, Australia; 2 School of Health Sciences, College of Health and Medicine, University of Tasmania, Launceston, TAS 7250, Australia; 3 MacKillop Family Services, South Melbourne, VIC 3205, Australia

**Keywords:** workplace, pregnancy, preconception, postpartum, community service, wellbeing, intervention mapping, co-design

## Abstract

Digital health interventions that specifically target working women across the preconception, pregnancy and postpartum (PPP) life stages may address the unique barriers to engaging in healthy lifestyle behaviours and self-care during this life phase. This paper describes the development of a workplace digital health intervention to promote healthy lifestyles and wellbeing for PPP women working at a community service organization in Australia. Intervention Mapping is a framework that guides program development, implementation, and evaluation. Steps 1 to 5 of Intervention Mapping methodology (needs assessment through to program implementation) were used, including identification of determinants and change objectives across socioecological levels (i.e., individual, interpersonal, and organisational) and iterative co-design and stakeholder engagement processes. The workplace digital health intervention was successfully developed and implemented as an online portal. Content included key strategies, information, and supports to promote health and wellbeing across PPP, including supporting the return to work in the postpartum period. Examples of resource pages included a parental leave checklist, process flows, Pride resources, and Aboriginal and Torres Strait Islander resources. Findings from a pilot feasibility study indicate the portal was accessible and beneficial for women in PPP life stages. The Intervention Mapping protocol may offer a valuable roadmap for collaborative design of interventions targeting PPP women’s behaviour and organisational work culture. Future work is needed to evaluate whether such interventions lead to improvements in women’s health and wellbeing.

## 1. Introduction

Preconception, pregnancy, and postpartum (PPP) are key life periods to promote healthy lifestyles and optimise wellbeing among women. Women of reproductive age (i.e., preconception stage) often have low adherence to recommended dietary and physical activity guidelines [1]. Half of all women of reproductive age from high-income countries are falling pregnant with a body mass index in the overweight or obese category [2], and approximately 50–60% of women exceed US National Academy of Medicine gestational weight gain recommendations during pregnancy [3]. Obesity in preconception is associated with increased risk of other chronic health conditions, including cardiovascular disease, Type 2 diabetes, high blood pressure, mental ill health and musculoskeletal conditions [4], which highlights the importance of promoting general preconception health [5]. Obesity in preconception and pregnancy is associated with increased risk of pregnancy complications and many adverse maternal and infant outcomes, including gestational diabetes [6], hypertension [6], miscarriage or still birth [6], pre-eclampsia [6], caesarean delivery [7], fetal macrosomia (i.e., large for gestational age) [7], and maternal psychopathology [8]. Difficulty returning to pre-pregnancy weight or experiencing additional weight gain in the postpartum period is also associated with negative maternal and child health outcomes, including increased cardiometabolic risks for both the mother and infant, maternal depression and body dissatisfaction, interpregnancy weight gain, risk of adverse maternal and infant outcomes for potential subsequent pregnancies, and increased risk of childhood obesity [9]. The link between lifestyle health and psychosocial wellbeing is well established [8]. Therefore, the importance of promoting healthy lifestyles and wellbeing before, during, and after pregnancy to facilitate healthy outcomes for mothers and their offspring is clear [8].

Preconception, pregnant, and postpartum working women face unique barriers to engaging in healthy lifestyle behaviours and self-care [10]. Preconception and pregnant working women have identified high workloads, competing responsibilities, job insecurity, discrimination, negative workplace norms and culture, policies, difficulty prioritising self-care, lack of time, and siloed departments as key work-specific barriers to health and wellbeing [11]. Conversely, work-specific facilitators to health and wellbeing include top-down workplace support and understanding, supportive relationships with colleagues, equity of access to healthy lifestyle opportunities, supportive physical and social workplace environment (i.e., design and setting), control over work scheduling, and flexibility in role and worker status [11]. Postpartum women also require tailored support in their transition to parenthood and return to work, and to prioritise their self-care and wellbeing during this period [12]. Enhancing the health of employees has also shown to significantly benefit the workplace at the organisational level, including reductions in absenteeism, presenteeism, compensation claims, and increased productivity and job satisfaction [13,14].

Workplaces present an ideal environment for promoting and enhancing employee’s health and wellbeing, and for delivering health education and knowledge (i.e., health promotion) to employees. A systematic literature review (led by members of this research team: SM, BH, HS) investigating the current state of knowledge regarding workplace lifestyle programs to improve diet, physical activity, and weight outcomes for working women [15] found workplace health interventions can be effective at improving lifestyle behaviours for women in the workplace [15]. Workplace lifestyle interventions and health promotion programs are associated with improvements in employee’s health and wellbeing outcomes, such as improved weight, nutrition, physical activity, and health information-seeking behaviour [13,15,16]. However, this review identified no programs targeted for PPP women that addressed their health needs in the preconception, pregnancy and postpartum periods.

Community service workplaces have been recognised as an opportune setting within which to promote the healthy lifestyles and wellbeing of PPP women [17]. There is a high proportion of PPP women in the community services workforce; females comprise approximately 80% of this workforce [18] and at least 70% of these women are of reproductive age [19]. Therefore, intervening in workplaces may provide a feasible and valuable opportunity to reach PPP women. This is particularly relevant for women who are vulnerable, for those who face barriers to accessing or engaging in traditional healthcare settings, such as preconception women (who often do not access health promotion information before awareness of pregnancy) [17], or postpartum women returning to work [20].

Workplaces have identified the value of and preference for digital health interventions [21]. Digital health interventions may overcome the established barriers to healthy lifestyle and wellbeing for PPP women via increased ease of access to information and support (i.e., available at anytime and anywhere, flexibility, not resource-intensive) to complement PPP women’s existing workload and other responsibilities [20,22]. However, there is currently an absence of literature regarding the existence of digital health interventions that specifically target working women across the PPP life stages [15]. There is a clear need for such interventions to be developed from the ground-up via stakeholder engagement, with the intended intervention target population group at the centre of the design and development process [23].

Intervention Mapping (IM) is a six-step program planning, implementation and evaluation framework that guides program designers to integrate theory, evidence, and data from the community [24,25]. Importantly, end-users and stakeholders contribute knowledge, experience, and practice at every step of IM to co-design new innovations that address significant health problems [24]. IM is underpinned by theoretical and evidence-based decision making, including behavioural change theory [26], an emphasis on co-design and participatory-based research, and an ecological systems approach to address the various individual, interpersonal, community, and societal factors that influence health and behavioural outcomes [24,25]. IM is well suited to guide the design and implementation of interventions in workplaces, which are inherently complex, multifaceted, and multi-layered settings [24]. It has been extensively applied to various health behaviour change and health promotion programs in workplace settings [27,28,29,30], and interventions focused on preconception [31], pregnancy [32], and postpartum [33] periods, albeit none with a focus on PPP women in the workplace. IM has also previously been used to develop digital health interventions to support the self-management of varied health concerns, including diabetes [34], back pain [35], and alcohol addiction [36].

Whilst a growing body of research has focused on examining the impact of digital health interventions in work settings, there are limited descriptions available of the intervention development process. To the authors’ knowledge, this is the first study to document the use of the IM methodology to support the health and wellbeing of PPP working women. Therefore, the aim of this paper is to describe the use of the IM methodology to develop a workplace digital health intervention (i.e., the Health in Planning, Pregnancy and Postpartum [HiPPP] online portal) to promote healthy lifestyles and wellbeing for PPP working women; our partner workplace is a community service organisation. This is the first digital health intervention tailored specifically for the workplace to the needs of PPP women.

## 2. Materials and Methods

Careful development of new interventions is necessary for programs to be adopted and effective in the real world. There are many published approaches to intervention development that rely on systematic processes, with program planners urged to draw on frameworks that are aligned to the purpose of the program [31,32,33]. IM was adopted due to its focus on behaviour change, guidance for integrating theory and research evidence, co-design approach with those who will use and implement the program, and extensive use in practice. The IM framework is a six-step iterative process, where each step builds on the decisions and products that are developed in the preceding steps [24]. The following sections summarise how the five IM steps were used to develop the workplace intervention, including: (1) logic model of the problem; (2) program outcomes and objectives; (3) program design; (4) program production; (5) program implementation plan; and (6) program evaluation plan. Table 1 presents the key project activities and outputs for each IM step; this paper focuses on Steps 1 to 5. A list of key IM terms and their definitions are provided in Supplement Table S1. Community participation is a key feature of the IM approach. Staff working across all levels of the community service organisation provided data that were used during each step.

### 2.1. Research Setting

Ethical approval was provided by the Monash University Human Research Ethics Committee (26934). Initial framing for this project was to develop a one-stop-shop web portal for the promotion of healthy lifestyles and wellbeing to support the prevention of maternal obesity across the key phases for the transition to parenthood (PPP).

The study was conducted in Melbourne, Victoria in collaboration with MacKillop Family Services (hereafter MacKillop)—a community service organisation that provides foster and residential out-of-home care, family support, alternative education, and disability services across Australia. MacKillop works to support and empower children, women, and families, with a focus on those experiencing family violence, disability, trauma, or other complexities. MacKillop has a predominantly female workforce, comprising 70% of their 1400 staff. They strive to be an employer of choice; to highlight and enhance their family-friendly culture; to assume responsibility to advocate for the wellbeing of the next generation; to nurture staff to improve the quality of their work and flow on benefits to the families they work with; to provide role models for young people and families, foster connection between female employees and the families they work with; and to provide information/resources for partners/fathers.

A project champion (DM) was appointed internally within MacKillop to oversee and facilitate the project between the researchers and the organisation. Researchers (SKM and/or BH) met weekly/fortnightly (as required) with the project champion via Microsoft Teams [37] to discuss project progress.

### 2.2. Step 1: Logic Model of the Problem

Step 1 involved examining the epidemiologic, behavioural, and social perspectives of the community at risk for health-related problems (PPP women), the intervention target population (PPP women employees), and the program setting (MacKillop) to create a logic model of the problem. A logic model of the problem is a graphic representation of the relationship between the health problems and their causes. It helps design groups to define the problem, the ecological levels that affect the problem, the multiple determinants of health-related behaviour and environment, and the stakeholders who will have insights on the problem and solution [24,38]. The IM framework guides program planners to develop the logic model of the problem by conducting a needs assessment that draws on multiple sources of data [24]. A systematic literature review, qualitative research with MacKillop staff (including employee survey), and discussion and workshopping with an advisory group and intervention design group, informed the logic model (see Table 1).

#### 2.2.1. Systematic Literature Review

As described above, a systematic review was conducted to examine workplace lifestyle programs to improve diet, physical activity, and weight outcomes for working women. Methods and results for the systematic review are reported elsewhere [15]. In brief, seven databases were searched for controlled studies that examined a workplace diet and/or physical activity intervention.

#### 2.2.2. Qualitative Research

Qualitative interviews and focus groups with MacKillop staff were conducted to understand the key needs, barriers, and facilitators to promoting health and wellbeing among PPP women at MacKillop. This qualitative research informed the program logic and program goals by providing an understanding of behavioural and environmental factors and determinants of wellbeing [11]. Methods and results for the qualitative interviews and focus groups are reported elsewhere [39]. Briefly, online interviews were conducted with 12 members of MacKillop’s executive team during December 2020–January 2021. Interview discussions explored participants’ work roles, perceived needs of PPP women, and barriers and facilitators to promoting the health and wellbeing of PPP women working at MacKillop.

Focus groups (*n* = 3; 14 employees) and individual interviews (*n* = 3) were conducted with 17 MacKillop PPP women employees, aged between 24 and 38 years, during February–March 2021 [39]. Table 2 presents the demographic characteristics of participants. The women worked across MacKillop’s family support services (50%), or education (18%), out-of-home care (18%) or disability services (12%). Discussions explored women’s perspectives of preconception, pregnancy, and postpartum health; health objectives and initiatives; work and health needs; and barriers and facilitators to health.

Interviews and focus groups were transcribed verbatim and thematically analysed. Two researchers independently coded the interviews, and discrepancies were resolved via comparison and discussion of code priorities to reach agreement. Codes were then grouped together to form overarching themes [39].

#### 2.2.3. Intervention Planning Groups

Two planning groups were established to guide the design, production, and implementation of the portal: an Advisory Group (*n* = 7) and a Design Group (*n* = 8). The Advisory Group consisted of seven senior executives and human resources (HR) representatives from MacKillop, and four researchers (SKM, AOC, CB, and HS). The purpose of the advisory group was to acquire input and guidance from senior employees and wellbeing champions throughout the design process. Advisory Group members were selected based on their role within the organisation and ability to support project implementation and success (e.g., access to HR, senior management) and invited to participate via email by the project champion (MacKillop HR). The Advisory Group met online via Zoom [40] or Microsoft Teams [37] for three meetings during the course of the project: following completion of the needs assessment (step 1), following completion of the co-design workshops (step 3), and prior to developing the program evaluation plan (step 6).

The design group consisted of eight staff working across the MacKillop organisation, including Property Service, Multisystemic Therapy, Internal Communications, IT, Child and Family Services, Education, and HR; and three researchers (SKM, AOC, CB). The purpose of the design group was to co-design the resources and activities that would form the HiPPP Portal. Staff from MacKillop with appropriate expertise or lived experience participated in the design group. This included women aged 18–45 years (given the focus on PPP women). Participants were recruited through staff newsletters and other MacKillop media (e.g., emails sent via HR). Participants who had previously participated in focus groups or staff interviews were also invited. Except for their knowledge or lived experience relating to healthy lifestyles and wellbeing for PPP women, participants were not required to hold specific skills or expertise. Three co-design workshops were conducted via Zoom [40] or Microsoft Teams [37] throughout the course of the project. Participants were also invited to provide written input on key outputs, such as change objectives, program goal, or workshop outputs via email. Table 3 presents the demographic details of the Advisory and Design Group participants. The majority of participants identified as female, 80% were born in Australia, 60% completed a Bachelor degree, 70% were employed full-time at MacKillop, and 70% were currently or had previously been a carer for children.

#### 2.2.4. Logic Model of the Problem and Program Goal

The first 2 h Design Group workshop (workshop 1) focused on Step 1—developing a logic model of the problem. Participants were given an overview of the qualitative needs assessment and were asked to discuss and prioritise the identified needs according to socio-ecological level (individual, interpersonal, and organisational) [25]. Participants were then asked to consider the causes or determinants, and context for these needs to inform the program goal. Participants also began to discuss the program goal but it was not finalised. To facilitate attendance and engagement, workshops 2 and 3 were one-hour sessions, following feedback from participants. During workshop 2, participants used the ‘how might we’ template [41] to continue refining the program goal using Mural [42], a digital whiteboard tool that facilitates online collaboration. Based on input from participants during workshop 2, four potential program goals were emailed to participants for review before workshop 3. The program goal was finalised at the beginning of workshop 3. The logic model of the problem was then finalised based on the co-design workshops and data generated through the needs assessment.

### 2.3. Step 2: Program Outcomes and Objectives

Step 2 focused on specifying detailed outcomes for the HiPPP online portal, which were guided by the socio-ecological model [25]. This theoretical model considers the complex interplay between an individual (e.g., personality characteristics, motivation, attitudes), the person’s relationships (e.g., relationships with co-workers), and broader community factors (e.g., work), which impact health and wellbeing [25]. Therefore, a behavioural outcome at the individual level, and environmental outcomes at the interpersonal level and organisational level were established by the researchers, based on information gained from Step 1. Each outcome was then subdivided into performance objectives. Performance objectives were defined as explicit behaviours required to achieve each behavioural and environmental outcome. The researchers identified change objectives by cross-tabulating performance objectives with the determinants identified during the first workshop in Step 1. Change objectives were operationalised as the change required to achieve the performance objective. A separate matrix of change objectives was created for each intervention level (i.e., individual, interpersonal, and organisational).

Draft program outcomes (at individual [behaviour], interpersonal and organisational [environmental] levels) were presented to design group participants during workshop 3. Following minor changes, participants agreed on program outcomes. The next step focused on developing performance objectives and change objectives. However, participants’ attendance at the design workshops varied greatly (i.e., participants often arrived late, left early, or did not attend at all). This was noted to be a common feature of the crisis orientation of the MacKillop workplace. 

#### Employee Survey

Given the need for additional staff input following workshop 3, an online 22-question survey (see Supplement Table S2) was disseminated to all MacKillop employees to identify program outcomes and objectives (including performance objectives and change objectives), and to inform program design and content. Survey questions were proposed by researchers based on findings from the needs assessment and initial workshops and agreed by the MacKillop project champion after consultation. A total of 120 employees completed the survey (8.57% of all employees); 91% were female, with a mean age of 39.04 years (ranging from 23 to 65 years). Average duration of employment at MacKillop was 3.83 years. Sixty-two percent of participants had children, with 25% intending to start or add to their family within the next two years. Survey results were analysed using R [43]. Based on the data collected through this survey and workshop discussions, the program goal was refined, and the determinants, program outcomes, performance objectives, and change objectives were identified.

### 2.4. Step 3: Program Design

The intervention was conceptualised and designed in Step 3, based on information gained from the qualitative needs assessment, survey, design group workshops and one-on-one sessions, and behaviour change theory. Due to time constraints, one-on-one sessions were conducted with six members of the intervention design group to generate broad ideas for program scope, themes, and delivery. Sessions ranged from 42 min to 1 h and were conducted online through Microsoft Teams [37]. Participants were asked to review the change objectives from Step 2 and consider strategies to improve the health and wellbeing of PPP women at MacKillop. Participants were encouraged to generate potential program ideas by workshopping the tools, skills, mind-set, and processes needed to achieve the program outcomes and objectives. This included program scope, components, materials, dosage, sequence, implementation, and delivery. Next, participants were asked to consider the appearance or design of the portal, including features, functions, colour, and platform. The researcher presented participants with examples of other workplace and/or healthy lifestyle resources targeting PPP women and were invited to comment on the visuals and content. Participants were also asked to comment on MacKillop’s staff intranet (the proposed location for the portal), including likes/dislikes, main usage, workflow, tools, suitability, and how well the proposed portal would integrate into the system. Based on input from the intervention design group, change methods (theory-based techniques to influence behaviour or environmental conditions) and practical strategies (to organise and operationalise the intervention methods) were identified.

### 2.5. Step 4: Program Production

The program content and materials were prepared in Step 4. Detailed program content related to workplace wellbeing, policies, and entitlements was developed internally by the project champion, in consultation with a representative from Payroll (as they are involved in leave process such as parental leave) and the Communication teams, based on the key findings from Step 3. The portal was integrated into MacKillop’s Intranet by the IT and Communications teams. Evidence-based resources relating to PPP health and wellbeing (i.e., fact sheets, links and videos) were supplied by the researchers.

Three design group participants were invited by the project champion to review an early version of MacKillop’s HiPPP Portal and provide initial feedback. The project champion and one researcher (SKM) demonstrated the draft portal and sought feedback during a one-hour session (online via Microsoft Teams [37]). The project champion also invited employees currently on parental leave to provide feedback on the portal via email. Content relating to Aboriginal and Torres Strait Islander People, LGBTQIA+ communities, and people living with disabilities were reviewed by appropriately trained staff and updated as necessary to ensure they were culturally safe and appropriate. By the end of Step 4, the HiPPP Portal had been designed and integrated into the MacKillop intranet.

#### Pilot and Feasibility Study

In January 2022, MacKillop staff were invited to participate in the HiPPP Workplace Portal pilot and feasibility evaluation to assess and refine the portal. The feasibility study aimed to determine whether the portal could support the health and wellbeing needs of all MacKillop employees by meeting the goals identified during the design process (described in the results). Staff were invited to complete a baseline survey, to use the portal over a one-month pilot period, and then complete a follow-up survey. The follow up survey included 39 questions about the extent to which staff found the portal easy to navigate and useful; 31 questions were answered on Likert scales, and 8 invited free text responses. Nine staff agreed to participate in the feasibility study, and six staff completed the follow-up survey.

### 2.6. Step 5: Implementation Plan

Step 5 focused on the creation of an implementation plan to encourage ongoing adoption and maintenance of the MacKillop HiPPP portal. A series of questions relating to implementation was drawn up by one researcher (SKM), in consultation with two other researchers (HS and CB), based on implementation literature, Consolidated Framework for Implementation Research (CFIR), and RE-AIM (Reach, Effectiveness, Adoption, Implementation and Maintenance, see Supplement Table S3). These were presented to the project manager in advance of a group meeting with HS, SKM and the project manager, HR director and member of the MacKillop communications team. The group discussed these questions during a 1.5 h long Microsoft Teams session. The final Advisory Group meeting was held to finalise the project and put the implementation plan in place to ensure ongoing uptake and monitoring of the portal. Development of a program evaluation plan is a longer-term goal and is beyond the scope of this paper.

## 3. Results

The following sections summarise the key outputs and findings that were produced using Steps 1–5 of the IM methodology, which contributed to the development of the HiPPP Portal.

### 3.1. Step 1: Logic Model of the Problem

Step 1 consisted of conducting a needs assessment, including a systematic literature review (findings of this review are reported elsewhere [15], qualitative review, and the development of a program goal and logic model of the problem.

#### 3.1.1. Systematic Review (Needs Assessment)

The findings of the systematic review are briefly described above and reported in detail elsewhere [15].

#### 3.1.2. Qualitative Research (Needs Assessment)

Findings of the qualitative interviews and focus groups are reported elsewhere and summarised in Table 4 [39]. Briefly, for the individual PPP women, key needs included coping skills, information and resources on diversity of needs and experiences, and support in managing expectations. At the interpersonal level, key needs were behaviour modelling of self-care, wellbeing, supportive culture, PPP open communication, and maintaining supportive relationships between staff. Needs at the organisational level included greater support for all PPP women, policies to support health of PPP women, greater education and awareness for managers, and enhancing the approach to PPP in high-risk environments [39].

#### 3.1.3. Logic Model of the Problem and Program Goal

**Figure 1:**
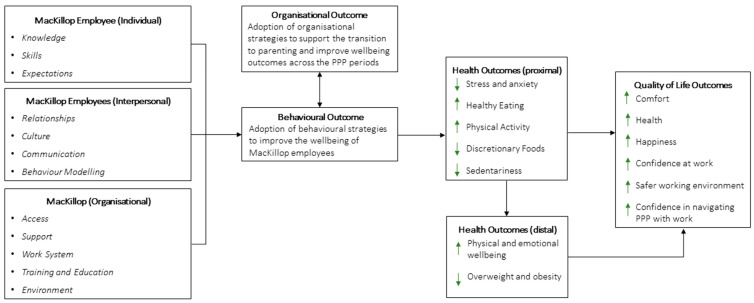
Logic Model of the HiPPP Portal.

The needs assessment informed the development of the logic model, as presented in Figure 1, that summarises the key determinants at each intervention level (individual, interpersonal and organisational), the behavioural and organisational outcomes, and health outcomes for MacKillop employees (proximal, distal, and quality of life outcomes). The overall goal of the HiPPP online Portal, as identified by the design group, was to support and understand the health and wellbeing needs of all MacKillop employees and encourage their safety, comfort, knowledge and belonging during all stages of PPP and parenting. Based on feedback and discussion in Advisory Group meeting 2, the portal direction was clarified to focus less on health outcomes and more about how the workplace can support holistic wellbeing during the PPP stages.

### 3.2. Step 2: Performance Outcomes and Objectives

The program outcomes and performance objectives for each socio-ecological level of intervention are presented in Table 5. A total of 16 performance objectives were identified across the individual, interpersonal, and organisation levels. The determinants for each socio-ecological level were established during workshop 1, informed by needs assessment data. These were cross-tabulated with the performance objectives to create matrices of change objectives, across each intervention level, and are presented in Supplement Tables S4–S6. Knowledge, skills, and expectations of PPP women were identified as determinants at the individual level. An example of a performance objective at the individual level was that MacKillop employees develop the skills to navigate available support and information platforms and improve their knowledge and capacity to engage with support. At the interpersonal level, relationships, culture, communication, and behaviour modelling were the key determinants of the behaviour of PPP women. At the organisational level, access, support, work systems, training and education, and organisational environment were considered key determinants to promoting health and wellbeing among PPP women at MacKillop.

### 3.3. Step 3: Program Design

The design group proposed an online resource embedded within the existing MacKillop staff intranet to meet the health and wellbeing needs of PPP women. Table 6 presents key components that cover the content, strategies, and design of the HiPPP portal, as identified by MacKillop staff from the design group workshop 3, one-on-one sessions, and the survey. Supplementary Table S7 links these strategies to the change objectives, determinants and theoretical underpinning, at each ecological level. Content areas were discussed and organised according to each life stage (i.e., preconception, pregnancy, postpartum and return to work) or the person’s role (i.e., parents, managers, and colleagues). Key strategies to deliver content included the provision of factual and clear information, healthy behaviour modelling, mentoring from fellow employees, access to support people external to the organisation (i.e., counselling), and opportunities for connection. MacKillop staff emphasised the importance of the portal being a one-stop-shop centralised platform; utilising an image-driven, easy access, simple language format; and following the existing online MacKillop style guide (e.g., page layouts, font type, size, and colours).

### 3.4. Step 4: Program Production

Step 4 consisted of developing and testing the draft the portal using the data gathered through steps 1–3.

#### 3.4.1. Program Development

**Figure 2:**
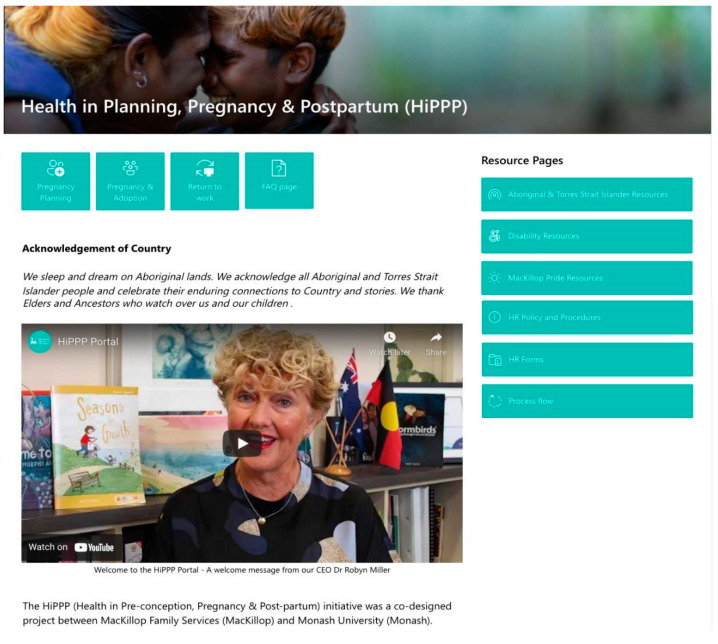
HiPPP Portal example webpage.

Figure 2 presents an example image of the HiPPP Portal. The portal covers health and wellbeing across three life stages: pregnancy planning; pregnancy; and return to work. Each section covers key strategies, information, and supports to promote health and wellbeing across each life stage, including support the return to work for women in the post-partum period. Key resource pages include a parental leave checklist, process flow, Pride resources, and Aboriginal and Torres Strait Islander resources.

#### 3.4.2. Pilot Feasibility Study

Of the six staff who completed the post-survey in the feasibility study, two staff used the portal 1–2 days per week, two staff members used the portal for 1–3 days over the month, and two staff used it once over the four weeks. Overall, participants agreed that the portal was easy to access, navigate, and understand. They also agreed that the HiPPP portal would be useful for both themselves, and others at MacKillop, particularly for those in the PPP life stages, and would encourage greater consistency of support and understanding from management and colleagues and contribute to a more supportive culture around wellbeing, health in PPP, and family needs at MacKillop. Supplement Table S8 presents detailed findings from the follow-up survey after one month of piloting the intervention.

### 3.5. Step 5: Program Implementation Plan

The Program Implementation Plan focused on organisation-specific mechanisms to deliver the HiPPP online portal through MacKillop’s existing online system while providing the relevant support needed to ensure its integration and ongoing sustainability. Implementation strategies included expression of interest (emails), CEO communication (email), Intranet articles, managers briefing (monthly), agenda items for RAP, MacKillop Pride, Disability Communities of Practice meetings, highlights poster (monthly), staff newsletter (bi-monthly), HiPPP posters and surveys. The program champion facilitated implementation within MacKillop, with support and guidance from the research team as needed. The portal is now currently available for all MacKillop staff to access at any time with their existing staff login details.

## 4. Discussion

This paper sought to describe the application of the IM methodology in the design and development of a workplace digital health intervention (named the HiPPP Portal) to promote healthy lifestyle behaviours and support wellbeing among PPP working women in a community service organisation. The HiPPP Portal was successfully developed and implemented at MacKillop through the use of IM. Key components included the comprehensive needs assessment to develop the program logic model, identification of determinants and change objectives across the socioecological levels of intervention (i.e., individual, interpersonal, and organisational), and iterative co-design and stakeholder engagement processes.

Meaningful stakeholder engagement and co-design are key strengths of this work. The two planning groups—the Advisory and Design groups—were crucial facilitators of the design, production, and implementation of the portal. The Advisory group ensured that the direction, focus, and suitability of the portal aligned with the vision, goals, and strategic direction of the organisation, and that appropriate supports could be implemented to develop the portal. The Design group ensured that the voices of the target population group (i.e., PPP women MacKillop employees) were represented in the design and development of the portal so that it accurately reflected their needs. This aligns with best practice participatory design research to utilise the skills, ideas, and lived experiences of the intervention target population, which is most likely to lead to sustainable change [26,35]. Indeed, all aspects of the HiPPP online portal (i.e., content, design, and usability) were directly shaped by the insights, experiences, and needs of MacKillop PPP women. This process also highlights the importance of a collaborative partnership between researchers and the industry partner to design and develop an intervention that can be embedded into standard organisational practice/systems. Researchers listened and acted upon any concerns identified by the MacKillop stakeholders, which further strengthened this partnership. Having a designated project champion from MacKillop also ensured that there was one consistent contact person to streamline communication, and the IM approach ensured that MacKillop were driving this work [24].

There are also key lessons to be learned from some of the challenges faced throughout the development process. This IM methodology was conducted throughout 2020–2021 in Melbourne, Victoria, amidst the height of COVID-19 and recurrent prolonged state-wide lockdowns. Consequently, all activities, meetings, design workshops and data collection related to this project were conducted online. Whilst this increased staff’s accessibility to participate, it also potentially limited the depth of discussions and engagement that could have been facilitated by face-to-face sessions. Relatedly, it was necessary to adapt the IM process to the nature of the workplace and priorities of employees. Community service organisations often support vulnerable or disadvantaged clients and families who are high-risk or in crisis (e.g., family violence situations). Given this nature of work, the online portal project was understandably a lesser priority among MacKillop employees and recruitment for the various activities was challenging. Hence, we took an adaptable approach to engaging with participants, for example swapping an additional design group workshop for a one-on-one interview, facilitating greater engagement and representation from MacKillop staff. Key barriers to implementing interventions and engaging with employees in the workplace often include lack of time, competing priorities, workplace culture, and the crisis-nature of some work types [44]. Whilst these factors presented challenges to the development of the online portal, they may also be the potential barriers to its ongoing implementation and uptake at MacKillop. Therefore, monitoring and evaluating the ongoing implementation and sustainability of the HiPPP online portal is underway.

The needs assessment and logic model of the problem drew upon a socio-ecological approach, ensuring needs across individual, organisational and interpersonal domains were addressed in the Portal design. While the program’s performance objectives are tailored to MacKillop to meet the needs of their employees, they may also be useful as a starting point when designing other workplace interventions. An example such as ensuring the workplace supports the safety and wellbeing of employees with family or HiPPP needs (performance objective 12) can be adapted to be more general. Indeed, socio-ecological theory was selected due to its relevance and applicability to the workplace setting [45]. Hence, while we encourage future workplace PPP wellbeing interventions to be co-designed to meet the specific needs of each organisation, we highlight the utility of our work as providing a foundational, clear, socio-ecological approach to inform future interventions. Public health practitioners, policy makers and workplaces can take several key lessons from this project. Firstly, as reiterated with the socio-ecological approach, addressing the wellbeing needs of working women requires input and support at all levels of management as well as via top down organisational support. Consequently, training and funding support targeting these needs is essential. Secondly, this work highlights the range and complexity of needs for working women across PPP. Hence, strategies to optimise women’s wellbeing must take into consideration this complexity. For example, healthcare practitioners seeing women for their wellbeing needs should consider work factors; policies impacting workplaces must incorporate women’s needs [46]; and workplaces must acknowledge women’s needs and those of their colleagues, incorporating training, access to resources, and appropriate leave policies.

The HiPPP portal is the first digital health intervention that has been specifically designed and tailored to the workplace and needs of PPP women. Nonetheless, there are some limitations of the developed intervention to acknowledge. First, as the HiPPP portal is an online information platform, it does not currently meet the need for in-person social support and connection to facilitate health and wellbeing, as identified by staff. Peer support and connection is a well-established facilitator of engagement in healthy lifestyle behaviours (i.e., exercise) for PPP women [9]. MacKillop is currently working on ways to address this and prioritise social support for PPP women in the workplace. Furthermore, while men are included and have access to the HiPPP portal, there is a heavy focus on the needs of women. Whilst this aligns with the key needs of Mackillop employees, supporting male partners and colleagues of PPP women is also crucial to facilitating health and fostering a supportive workplace culture around HiPPP and family needs [8,9,10]. Finally, there was a stronger focus on the pregnancy and postpartum life stages compared with preconception. This was due to preconception health and wellbeing featuring less often in the needs assessment. This may be due to various factors, including lack of pregnancy planning or the desire to keep pregnancy planning a secret [5]. Nonetheless, the portal reflects the needs identified by the employees. MacKillop also successfully addressed participants’ early concerns and considerations about the functionality of the HiPPP Portal throughout the design and development process. As one part of the MacKillop intranet, there was the risk that the HiPPP Portal may be “lost” among other content across the site or difficult to locate. Therefore, the HiPPP tile was predominately located on the intranet’s home landing page in a prominent position. Further, the nature of the online portal ensured employees’ privacy and confidentiality as staff could access the portal privately if necessary.

## 5. Conclusions

This paper describes the successful development of the MacKillop HiPPP Portal using the IM methodology to enhance the health and wellbeing of MacKillop employees during pregnancy planning, pregnancy and postpartum; to our knowledge, it is the first intervention specifically targeting PPP working women. The comprehensive process enabled a multi-disciplinary team to develop an intervention based on theory and evidence. The findings suggest the IM protocol may offer a valuable roadmap for PPP health researchers, PPP women, and community service workplaces to design interventions that target PPP women’s behaviour and organisational work culture, with potential utility in informing future intervention design and policy change for PPP working women’s wellbeing. Future work is needed to evaluate whether the implementation of such interventions in the workplace can yield significant improvements in maternal health and wellbeing.

## Figures and Tables

**Table 1:** IM steps, research activities and outputs.

IM Step	Research Activity	Outputs
Step 1. Logic Model of the Problem	Establishment of the planning groups	Key needs of employees
	Systematic review	Program goal
	Qualitative research with executives & employees	Logic Model of the problem
	Design group workshops 1, 2 and 3	
	Employee survey	
Step 2. Program Outcomes and Objectives	Employee survey Design Group workshop 3	Program outcomes and performance objectives
		Matrices of change objectives
Step 3. Program Design	1:1 design group interviews	Content, design and strategies for the HiPPP Portal
	Employee survey	
Step 4. Program Production	Pilot and feasibility studyReview and feedback sessions	HiPPP portal embedded into workplace Intranet
Step 5. Program Implementation Plan	Development of implementation plan and strategies	Implementation of the portal

**Table 2:** Demographic characteristics of MacKillop employee interview and focus group participants.

Variable	Participants (*n* = 16) *
Mean age (range), years	31.5 (24–38)
Has children, *n* (%)	12 (75)
Number of children	
Mean (range)	1.4 (1–2)
Median	1
Employment status, n (%)	
Full-time	11 (69)

* One participant did not complete the demographic questionnaire.

**Table 3:** Demographic characteristics of the intervention planning groups.

Variable	Advisory Group Participants	Design Group Participants
Female sex, *n* (%)	6 (83%)	4 (50%)
Mean age (range), years	53.3 (47–63)	34 (28–37)
Mean duration of employment at MacKillop (range)	4 years 3 months (1 year 7 months–6 years 4 months)	3 years 6 months (7 months–7 years 4 months)

**Table 4:** Prioritising the needs from the needs assessment.

Individual	Interpersonal	Organisational
*Skills* Coping skills, e.g., managing feelings of guilt about pregnancy	*Behaviour modelling* Top-down behaviour modelling of self-care and wellbeing Model open communication to new staff	*Data Policy* Inclusivity Clarify entitlements Improve navigation of policies Miscarriage support
*Diversity of needs and experiences* Same-sex partnerships Gender diversity Aboriginal and Torres Strait Islander People Illness and depression during and after pregnancy	*Culture* Foster supportive and open environment for pregnancy	*Support for all PPP women* Pregnancy IVF Grief counselling Miscarriage and stillbirth Adoption Pregnancy-related depression Return to work
*Expectations* Individual expectations of PPP needs and supports Unique to individual	*Communication* Normalise and increase communication about early pregnancy Promote open communication of needs throughout pregnancy	*Education* Education and awareness for managers
		*Environment* Physical violence and high-risk environments (i.e., family violence situations) Approach to PPP in high-risk environments

**Table 5:** Program Outcomes and Performance Objectives for the HiPPP Portal According to Socio-Ecological Level.

Program Goal	Level	Program Outcome	Performance Objective (PO)
To support and understand the health and wellbeing needs of all MacKillop employees and encourage their safety, comfort, knowledge, and belonging during all stages of HiPPP and parenting	MacKillop Employee (Individual)	MacKillop employees feel informed, valued, safe, and supported with their family, health, and wellbeing needs throughout their HiPPP and parenting journey	PO1: MacKillop employees build a knowledge of the supports available to them at MacKillop to support their family and HiPPP needs PO2: MacKillop employees build confidence that their HiPPP needs will be met and understood PO3: MacKillop employees develop the capacity and skills to engage with supports PO4: MacKillop employees understand of their PPP rights and responsibilities PO5: MacKillop employees develop the skills and knowledge to manage their PPP, caring, and health needs with work PO6: MacKillop employees develop skills and knowledge to manage changes to working conditions brought about by COVID
	MacKillop Employees (Interpersonal)	MacKillop employees develop an understanding of the needs of those with families or those wanting to start or add to their family and facilitate connection and open communication of those needs	PO7: Colleagues and managers provide support and understanding for the needs of families or those with HiPPP needs PO8: Managers develop the knowledge and skills to support wellbeing, family, and HiPPP needs PO9: MacKillop employees foster open communication and a supportive culture around HiPPP and family needs PO10: MacKillop employees keep in touch with those on parental leave and support their transition back to work PO11: MacKillop employees connect with those in a similar life stage
	MacKillop (Organisational)	MacKillop endorses employee-centred strategies to support and normalise wellbeing, HiPPP, and parenting needs, and ensures structured support and understanding is provided across all roles	PO12: MacKillop supports the safety and wellbeing of employees with family or HiPPP needs PO13: MacKillop ensures all employees can access supportive wellbeing, family and HiPPP-related information, e.g., parental leave policy PO14: MacKillop facilitates integration of specific supports, policies, and/or procedures to normalise families and HiPPP needs in the workplace PO15: MacKillop supports staff to work through challenging and changing conditions (COVID) PO16: MacKillop ensures that employees have adequate supports and resources to meet their work demands

**Table 6:** Examples of content, strategies, and design for the HiPPP Workplace Portal at MacKillop.

Content	Strategies	Design
*Preconception* IVF Diet and exercise guidance before pregnancy Sex-specific health conditions, e.g., dysmenorrhea	*Information provision* Trustworthy, factual, and clear Fact sheets, videos, flow charts, box pops, personal stories	One-stop-shop Centralised platform Dynamic and frequently updated Simple language Accessible Image driven
*Pregnancy* Work safety Appointments, e.g., antenatal Pregnancy and loss	*Behaviour Modelling* Top-down modelling of self-care, wellbeing, and boundaries Communication from senior executives demonstrating support	Inclusive and supportive of diversity Follow the MacKillop style guide Sectioned according to life stage
*Postpartum and the return to work* Building confidence in the employee’s ability to return to work Breastfeeding Postpartum depression	*Mentoring* List of employees who can provide support to others trying to manage PPP and parenting	
*Parents* Adoption and fostering Supports for single mothers Workplace flexibility	*Access to support person* Unrelated to employee’s team or operations	
*data Manager* Role guidance around preconception, pregnancy, and postpartum (PPP) Available supports for PPP employees	*Counselling* Grief and loss support Access to employee assistance program Options for debrief	
*Colleagues* How to support pregnant colleagues How to keep in touch with those on leave Responding to a colleague’s pregnancy	*Space for connection* Opportunity to connect with others in a similar life stage Keep in touch options during leave Regular meetings to provide support to families and partners	
*Other* MacKillop expectations during PPP MacKillop vision and values for pregnancy, parents, and families	*For managers and HR* Training and education Scheduled catch ups and check ins	

## Data Availability

Given the nature of the data and its link to a workplace, data is not available in a publicly archived dataset. Queries may be directed to the corresponding author.

## Supplementary Materials

The following supporting information can be downloaded at: https://www.mdpi.com/article/10.3390/ijerph192215078/s1, Table S1: Intervention Mapping terms and definitions; Table S2: Survey distributed to MacKillop employees; Table S3: Implementation Plan Questions; Table S4: Matrix of change for MacKillop employees (individual level); Table S5: Matrix of change for MacKillop employees (interpersonal level); Table S6: Matrix of change for MacKillop employees (organisational level); Table S7: Overview of change objectives mapped to theory-based methods and example strategies; Table S8: Feasibility study findings.
