# Supplementary material for: Using Intervention Mapping to Develop a Workplace Digital Health Intervention for Preconception, Pregnant, and Postpartum Women: The Health in Planning, Pregnancy and Postpartum (HiPPP) Portal
Source: Int J Environ Res Public Health. 2022 Nov 16;19(22):15078. doi: 10.3390/ijerph192215078 (PMC9690929; doi:10.3390/ijerph192215078)
Supplement: Supplementary file 1 [file ijerph-19-15078-s001.zip › ijerph-1988154-supplementary.pdf]

International Journal of
Environmental Research and Public Health

MDPI

Using Intervention Mapping to develop a workplace digital health intervention for preconception, pregnant and postpartum women: The Health in Planning, Pregnancy and Postpartum (HiPPP) Portal

Supplementary Material

Claire Blewitt [1,†], Melissa Savaglio [1,†], Seonad K. Madden [1,2], Donna Meechan [3], Amanda O'Connor [1], Helen Skouteris [1] and Briony Hill [1,*]

1 Health and Social Care Unit, School of Public Health and Preventive Medicine, Monash University, Melbourne, VIC 3004, Australia
2 School of Health Sciences, College of Health and Medicine, University of Tasmania, Launceston, TAS 7250, Australia
3 MacKillop Family Services, South Melbourne, VIC 3205, Australia
* Correspondence: briony.hill@monash.edu
† These authors contributed equally to this work.

Supplement Table S1

Intervention Mapping terms and definitions

**Intervention Mapping Term**	**Definition**
Change objectives	Specific changes in behaviours that are proposed based on their determinants
Determinants	Factors that influence/contribute to the health problem(s)
Intervention Mapping	A protocol/framework for developing theory-based and evidence-informed interventions
Logic model of the problem	A graphic representation of the relationship between the targeted health problem(s) and their causes
Needs assessment	A systematic process to determine the priorities needs of the target group, or gaps between the current situation and the desired situation, to inform program goals
Performance objectives	The specific changes that need to occur to achieve the identified outcomes

Supplement Table S2

Survey distributed to MacKillop employees

#	Question	Question Style
	Demographics	
1	What is your sex?	Multiple Choice
2	What is your gender?	Multiple Choice
3	What is your age in years?	Short Answer
4	Please state your country of birth:	Short Answer
5	Are you of Aboriginal or Torres Strait Islander origin?	Multiple Choice
6	Where is your workplace located?	Multiple Choice
7	Which of these best describes your work at MacKillop?	Multiple Choice
8	How long have you worked at MacKillop?	Short Answer
9	What is the composition of your household?	Multiple Choice
10	Do you have any children?	Multiple Choice
10b	If yes, how many?	Multiple Choice
10c	What is the age of the youngest child in your care?	Multiple Choice
11	Are you planning to start or add to your family in the next 12 months?	Multiple Choice
12	Do you provide care to a family member, partner, or friend who needs support due to illness, frailty, disability, or addiction?	Multiple Choice
13	What is the highest level of education you have completed?	Multiple Choice
	Work and Wellbeing	
14	Are there any aspects of work that impact your health and wellbeing?	Tick Box
	Tick all that Apply	
	Relationship with my team or manager	
	Knowledge (e.g. knowing about EAP)	
	Work demands, e.g. heavy workloads	
	High-stress and/or high-risk environment	
	Policies, entitlements and/or procedures, e.g. return to work	
	Work expectations, e.g. responding to crises on day off	
	Access to parenting amenities, e.g. breastfeeding/pumping spaces	
	Access to wellbeing initiatives or healthy food, e.g. yoga class	
	Managing health conditions, e.g. dysmenorrhea, with work	
	Managing pregnancy experiences (incl. miscarriage, IVF, early pregnancy) with work	
	Managing parenting with work	
	Top-down support	
	Team activities, e.g. morning teas and informal meet ups	
	Access to flexible work arrangements	
	Connecting with other parents at MacKillop	
	Managers having the necessary skills and information to provide guidance and support	
	Other: ______________________________	
14b	Additional Comments	Short Answer
15	Are you aware of any current MacKillop supports to help with your health and wellbeing needs during the preconception, pregnancy, or postpartum periods?	Multiple Choice
15b	If yes, what supports are available?	Short Answer
15c	Do you feel you have access to these supports?	Multiple Choice
	Health in Preconception, Pregnancy and Postpartum (HiPPP) Portal	
16	Are you aware of the HiPPP project in MacKillop?	Multiple Choice
17	If yes, what would you like this project to achieve?	Short Answer

18	What specific outcomes would you like to see (e.g. improved health, social connection, or wellbeing)?	Short Answer
19	Who is this program for?<br>*Tick all that apply*<br>*Women going through the preconception, pregnancy, or postpartum (PPP) life phases*<br>*Partners going through the PPP life phases*<br>*Same sex partners going through the PPP life phases*<br>*Gender diverse partners going through the PPP life phases*<br>*Parents*<br>*Grandparents (employees)*<br>*Colleagues*<br>*Managers*<br>*Other, please specify: ____________________*	Tick Box
20	What would you like to see on the portal?<br>*Tick all that apply*<br>*How to manage pregnancy and work requirements*<br>*Arranging pregnancy appointments during work hours*<br>*Support for women going through miscarriage or IVF*<br>*Clear policies and entitlements, e.g. parental leave*<br>*Information about breastfeeding/pumping at work*<br>*What health and wellbeing supports are available at MacKillop, e.g. EAP*<br>*How to navigate EAP and what to expect*<br>*How maternity leave affects other entitlements (e.g. long service leave)*<br>*Connect to employee mentoring to help navigate work and the PPP and parenting life phases*<br>*Template application libraries, e.g. what an application for flexible work arrangements looks like*<br>*Opportunities for connection while on parental leave*<br>*Connect with other mothers or women in the preconception/pregnancy phase*<br>*Diet and exercise guidance before and after pregnancy*<br>*Build confidence and support for the return to work*<br>*Guiding information for dealing with staff pregnancy and client grief, e.g. client miscarriage*<br>*MacKillop's vision and values for pregnancy, parents, and families*<br>*Points of contact, i.e. who to contact for support or information at MacKillop*<br>*Example scenarios, e.g. how might pregnancy affect your work role?*<br>*Support and information for partners*<br>*What to expect at MacKillop during the preconception, pregnancy, and postpartum phases*<br>*Inclusive language and information, e.g. gender diversity or sexual orientation*<br>*How to have conversations with your supervisor around pregnancy or parenting, e.g. what questions should you ask?*<br>*Basic pregnancy-related information, e.g. vaccinations, cervical screening*<br>*Counselling support*<br>*Links to parenting and maternal health supports*<br>*Dealing with loss and grief, or health problems arising from pregnancy or birth at MacKillop*<br>*Other, please specify: ____________________*	Tick Box
21	Any additional comments?	Short Answer

Supplement Table S3
Implementation and evaluation questions

#	Question
	Reach and Adoption
1	Who will benefit from the HiPPP Portal?
2	How will you know you have reached the target groups?
3	What adoption outcomes do we wish to achieve and what do we need to do to achieve them?
4	What are the barriers and enablers to adoption of the HiPPP Portal (individual and organisational level)?
5	How well will the HiPPP Portal 'fit in' at MacKillop? Are any steps being taken to improve the fit? (e.g., alignment with organisational goals and priorities, alignment with MK culture and values etc.)
6	What steps will be taken to improve acceptance and adoption of the HiPPP Portal?
	Implementation
7	What implementation outcomes do we wish to achieve?
8	What do we need to do to achieve them?
9	Who are the key employees involved in the implementation of the HiPPP Portal?
10	Who do you need to consult?
11	What are the barriers and enablers to implementation of the HiPPP Portal? (individual and organisational level)
12	Is there an ideal time to implement the HiPPP Portal at MacKillop? (e.g., leadership engagement, resources, access)
13	Whose buy-in, input, and expertise is needed for success? (e.g., approvals, skills, resources)
14	Will the program require different people to implement different components?
15	What key supports and strategies are needed to deliver the program at MacKillop? Please describe.
16	Why are these supports and strategies the most important? (e.g., costs, strategic plan)
17	Are any infrastructure changes needed to accommodate the overall program, e.g., breastfeeding supports?
18	Will you use guiding documents or information to facilitate your implementation strategy?
19	Does anything need to change to implement the portal/program (from usual procedure or processes)?
20	How will MacKillop assure that the program is delivered properly, i.e. as intended, and consistently, i.e., according to the needs and strategies identified during the needs assessment and workshops?
21	Are there/have there been any unforeseen costs or factors associated with implementation of the HiPPP Portal?
22	How will implementation be evaluated?
	Maintenance
23	What are the barriers and enablers to maintenance of the portal (individual and organisational)?
24	How will the portal be maintained over time, so that it continues to be effective and delivered as designed?
25	Who will maintain the portal? (e.g., updates or changes)
26	How will maintenance be monitored?

	Evaluation
27	How will we know the portal has achieved its goal? (Program goal: To support the health and wellbeing of all MacKillop employees (including partners) and encourage their safety, comfort, knowledge, and belonging before and after pregnancy, and when starting a family)
28	How might we measure the success of the program? (e.g., pre- and post-questionnaire for pilot participants and/or tracking portal engagement)
29	Can usage or outcomes of the portal be monitored? Can this be linked to data already being collected at MacKillop? What data is currently being collected?

Supplement Table S4

Matrix of Change Objectives for MacKillop Employees (Individual Level)

Performance Objectives (PO)	Determinants		
	Knowledge (K)	*Skills (S)*	*Expectations (E)*
PO1: MacKillop (MK) employees build a knowledge of the supports available to them at MacKillop to support their family and Health in Planning, Pregnancy, and Postpartum (HiPPP) needs	K1.1 MK employees can identify available resources or supports to meet their family, PPP, or wellbeing needs at work<br>K1.2 MK employees are aware of which supports are available to them according to their work role (e.g., casual status)<br>K1.3 MK employees know how parental leave affects other entitlements	S1.1 MK employees feel confident that they can manage their pregnancy appointments with work	E1.1 Employees expect that supports accommodating family and HiPPP needs are available<br>E1.2 Employees know what to expect at the workplace during PPP
PO2: MK employees build confidence that their HiPPP needs will be met and understood	K2.1 MK employees know who to contact to have their needs met and understood	S2.1 MK employees develop the confidence to advocate for their needs<br>S2.2 MK employees can explain their needs to colleagues and management<br>S2.3 MK employees are confident they can manage the return to work	E2.1 MK employees expect they will be comfortable when returning to work
PO3: MK employees develop the capacity and skills to engage with supports	K3.1 MK employees know where to find information to help develop the skills to engage in supports	S3.1 MK employees develop the skills to navigate available support and information platforms	E3.1 MK employees expect training or information will be provided to help navigate the MK system
PO4: MK employees understand of their PPP rights and responsibilities	K4.1 MK employees understand workplace expectations when they return to work<br>K4.2 MK employees know where to find information regarding their rights and responsibilities		E4.1 MK employees expect that their rights and responsibilities will be available in a clear and relevant manner.

PO5: MK employees develop the skills and knowledge to manage their PPP, caring, and health needs with work	K5.1 MK employees know where to seek help if they are struggling to manage their needs with work	S5.1 MK employees develop their self-efficacy to manage their needs with work	E5.1 MK employees expect that there will be period of adjustment and support following the return to work<br><br>E5.2 MK employees expect that they will be supported to manage their needs with work
PO6: MacKillop employees develop skills and knowledge to manage changes to working conditions brought about by COVID	K6.1 MK employees know where to access supports and resources to manage working from home<br><br>K6.2 MK employees are aware of workplace expectations during challenging conditions such as the COVID pandemic<br><br>K6.3 MK employees are aware of physical and mental health supports to help them manage during COVID	S6.1 MK employees develop the self-efficacy to manage challenging and changing circumstances with work	E6.1 MK employees expect the workplace will adjust expectations and make accommodations during COVID

Supplement Table S5

Matrix of Change Objectives for MacKillop Employees (Interpersonal Level)

Performance Objectives (PO)	Determinants			
	Relationships (R)	*Culture(C)*	*Communication (COMM)*	*Behaviour Modelling (BM)*
PO7: Colleagues and managers provide support and understanding for the needs of families or those with Health in Planning, Pregnancy, and Postpartum (HiPPP) needs	R7.1 Managers and staff manage their work expectations of those with HiPPP or parenting needs<br>R7.2 Managers and colleagues are inclusive of those with families or HiPPP needs in organising relationship- or team-building activities	C7.1 Managers understand the importance of leading and guiding their employees through the PPP and family space<br>C7.2 Managers and colleagues actively take steps to greater understand and support those with family of HiPPP needs	COMM7.1 Managers and colleagues encourage open communication of needs<br>COMM7.2 Managers communicate entitlements more broadly	BM7.1 Managers and Colleagues model supportive and understanding behaviours in the workplace<br>BM7.2 Managers model wellbeing behaviours (e.g., not working through lunch) to their team
PO8: Managers develop the knowledge and skills to support wellbeing, family, and HiPPP needs	R8.1 Managers ask their staff how they can best support their needs	C8.1 The skills and knowledge to support wellbeing, family, and HiPPP needs are automatically part of the manager skillset<br>C8.2 Managers use their knowledge and skills to facilitate a supportive culture	COMM8.1 Managers develop the knowledge and skills to communicate support of needs effectively	BM8.1 Managers impart knowledge and skills to their senior team members<br>BM8.2 Managers demonstrate knowledge and skills during team meetings
PO9: MacKillop (MK) employees foster open communication and a supportive culture around HiPPP and family needs	R9.1 MK employees ask those with HiPPP and family needs how they can provide support	C9.1 MK employees contribute to a supportive HiPPP and family culture		BM9.1 MK employees share their own experiences of HiPPP and family needs<br>BM9.2 MK employees may act as mentors to those needing guidance around HiPPP, parenting and work

PO10: MK employees keep in touch with those on parental leave and support their transition back to work	R10.1 MK teams maintain their relationships with those on leave	C10.1 Inclusion of those on parental leave in work opportunities, updates, or activities is a normal part of team culture<br><br>C10.2 Teams welcome back those who have returned to work and help them readjust	COMM10.1 Managers gather personal emails for those going on parental leave to maintain contact<br><br>COMM10.2 Managers organise regular online catchups or lunch and learns are available to parents on parental leave<br><br>COMM10.3 Managers communicate when staff are returning to work to the rest of the team	BM10.1 Managers contact employees on leave and provide their team with updates
PO11: MK employees connect with those in a similar life stage	R11.1 MK employees connect with others at MK who have similar family or PPP needs<br><br>R11.2 MK employees foster team relationships outside of work<br><br>R11.3 Mk employees engage in team-building activities at work	C11.1 MK employees regularly organise opportunities to connect with others in a similar life stage	COMM11.1 A communication or chat forum is established for MK employees to connect and communicate during the PPP and family life stages	BM11.1 MK employees tell others about the benefits of connecting with others at MK<br><br>BM11.2 Teams regularly attend team- or relationship-building activities

Supplement Table S6

Matrix of Change Objectives for MacKillop (Organisational Level)

**Performance Objective (PO)**	**Determinants**				
	Access (A)	*Support (SU)*	*Work System (WS)*	*Training and Education (TE)*	*Environment (ENV)*
PO12: MacKillop (MK) supports the safety and wellbeing of employees with family or Health in Planning, Pregnancy, and Postpartum (HiPPP) needs	A12.1 MK provides access to wellbeing supports for those with HiPPP and family needs<br><br>A12.2 MK provides time for employees to attend pregnancy appointments	SU12.1 MK communicates its vision and values for employee safety and wellbeing, specifically for those with family and wellbeing needs<br><br>SU12.2 MK supports employees to manage pregnancy and work requirements<br><br>SU12.3 MK provides specific support for those going through IVF<br><br>SU12.4 MK provides specific support for those experiencing grief and loss, e.g., miscarriage	WS12.1 MK ensures work processes do not put an excessive work burden on any employee	TE12.1 MK provides training and education opportunities to all employees to facilitate understanding of family and HiPPP needs<br><br>TE12.2 MK provides education on safety concerns and responsibilities, particularly during PPP<br><br>TE12.3 MK provides education for managers to support their team members through grief and loss	ENV12.1 MK provides role flexibility and opportunities for women to ensure their safety during pregnancy<br><br>ENV12.2 MK cultivates a safe environment for all employees
PO13: MK ensures all employees can access supportive wellbeing, family and HiPPP-related information, e.g. parental leave policy	A13.1 Policies and information are located in a centralised position (HiPPP Portal) and are easy for staff to access and navigate<br><br>A13.2 Policies are clear for all staff to understand	SU13.1 Policies to support families or those with PPP needs are changed to use more inclusive language<br><br>SU13.2 MK provides employees with the opportunity to seek	WS13.1 MK provides the necessary support structures (e.g. SharePoint, allocation of meeting time) to facilitate information exchange WS13.2 MK facilitates an environment where employees feel	TE13.1 MK provides new and returning employees with an induction detailing how to access information<br><br>TE13.2 MK team leaders provide regular updates and reminders of how to access information	

		information during work time	comfortable accessing information	TE13.3 MK provides information to employees about breastfeeding or pumping at work	
PO14: MacKillop facilitates integration of specific supports, policies, and/or procedures to normalise families and HiPPP needs in the workplace	A14.1 MK improves access to contact days during parental leave<br><br>A14.2 MK puts a formalised process in place to assist the return to work<br><br>A14.3 MK facilitates equitable access to parenting amenities for employees, e.g., pumping spaces	SU14.1 Parental leave policies are adapted to include the return to work<br><br>SU14.2 MK supports PPP and parenting employees to manage their parenting, pregnancy experiences, elder care responsibilities, and health conditions with work<br><br>SU14.3 Supports are suited to meet the diversity of needs and experiences of MK employees with families or during the PPP periods, e.g., IVF, miscarriage, stillbirth	WS14.1 MK endeavours to ensure that policies, supports, and procedures align in practice	TE14.1 MK provides clear information on supports, policies, and procedures to all staff<br><br>TE14.2 MK provides training for managers to understand the need for flexible work changes, education on policies, employee experiences, and proving a safe environment for employees<br><br>TE14.3 MK includes partners in HiPPP and parenting communications and information	ENV14.1 MK provides a supportive environment for employees to voice their HiPPP and family needs
PO15: MK supports staff to work through challenging and changing conditions (COVID)	A15.1 MK provides access to appropriate working from home supports<br><br>A15.2 MK provides access to physical and mental health supports	SU15.1 MK provides extra support to those struggling with family demands and work during COVID	WS15.1 MK ensures that current work processes are equitable for employees during COVID	TE15.1 MK provides education to managers on the best methods to keep staff informed and supported during COVID, particularly for those with PPP or family needs	ENV15.1 MK ensures that staff who need to work from home are not disadvantaged compared to those ‘at work’

PO16: MK ensures that employees have adequate supports and resources to meet their work demands	A16.1 MK provide access to adequate resources to meet work demands	SU16.1 MK ensures that those returning to work have adequate support to transition back to work at an appropriate pace	WS16.1 Mk ensures work demands are not excessive and are adaptive to employee capacity	TE16.1 MK facilitates employee handover prior to parental leave	

Supplement Table S7

Overview of Change Objectives Mapped to Theory-Based Methods and Example Strategies

Determinant	Targeted Change Objectives	Method (Theoretical Basis)	Example Strategies
Individual Level			
Knowledge	K1.1, K1.2, K1.3, K2.1, K3.1, K4.1, K4.2, K5.1, K6.1, K6.2, K6.3	Consciousness raising (HBM)	Fact sheets, flow charts, videos, box pops, personal stories
	K1.1, K4.2, K6.1	Imagery (TIP)	Fact sheets, box pops, videos
	K1.1, K1.2, K1.3, K4.1	Tailoring (TTM)	Fact sheets, flow charts, videos, box pops, personal stories. Sectioned according to life stage and supportive of diversity
Skills	S1.1, S2.1, S2.3, S5.1, S6.1	Modelling (SCT)	Videos, personal stories
	S1.1, S2.1, S2.2, S2.3	Persuasive Communication (ELM)	Fact sheets, flow charts, videos, box pops, personal stories
	S1.1, S2.1, S3.1, S5.1, S6.1	Active Learning (ELM)	Fact sheets, flow charts, videos, box pops, personal stories
Expectations	E1.1, E1.2, E2.2, E5.1, E5.2, E6.1	Cultural similarity (CPM)	Videos, personal stories
	E1.1, E1.2, E2.2, E3.1, E4.1, E5.1, E5.2, E6.1	Elaboration (ELM, TIP)	Fact sheets, Flow charts, videos, box pops, personal stories
Interpersonal Level			
Relationships	R7.1, R7.2, R8.1, R9.1, R10.1, R11.1, R11.2	Elaboration (ELM, TIP)	Communication from senior staff<br>Scheduled catch ups and check ins<br>Keep in touch options<br>Regular meetings to provide support
	R11.1	Cultural Similarity (CPM)	Opportunity to connect with others in a similar life stage<br>Mentoring
	R7.2	Modelling (SCT)	Top-down modelling
	R8.1, R9.1, R10.1, R11.2, R11.3	Reinforcement (TL, SCT)	Scheduled catch ups and check ins<br>Regular meetings to provide support<br>Top-down modelling

	R10.1, R11.1, R11.2, R11.3	Interpersonal Contact (TSD)	Opportunity to connect with others in a similar life stage<br>Keep in touch options<br>Scheduled catch ups and check ins
Culture	C7.1, C7.2, C8.2, C9.1	Modelling (SCT)	Top-down modelling<br>Training and education
	C10.1, C10.2, C11.1	Interpersonal Contact (TSD)	Keep in touch options<br>Regular meetings to provide support<br>Opportunity to connect with others in a similar life stage
	C7.1, C7.2, C8.1	Training (TSD, SCT)	Training and education<br>Communication from senior staff
Communication	COMM7.1, COMM10.1, COMM10.2, COMM10.3, COMM11.1	Mobilising social support (DIT)	Top-down modelling<br>Regular meetings to provide support<br>Keep in touch options<br>Opportunity to connect with others in a similar life stage
	COMM7.1, COMM7.2, COMM10.1	Discussion (ELM)	Regular meetings to provide support<br>Scheduled catch ups and check ins
	COMM10.3, COMM11.1<br>COMM8.1	Training (TSD, SCT)	Training and education
Behaviour Modelling	BM7.1, BM7.2, BM8.1, BM8.2, BM9.1, BM9.2, BM10.1, BM11.1, BM11.2	Modelling (SCT)	Top-down modelling<br>Training and education<br>Keep in touch options<br>Communication from senior staff<br>Regular meetings to provide support
Organisational Level			
Access	A12.11, A12.2, A13.1, A13.2, A14.1, A14.2, A14.3, A15.1, A15.2, A16.1	Facilitation (SCT)	Access to support person<br>Grief and loss support<br>Options for debrief<br>Communication from senior staff<br>Access to employee assistance program

	A12.2	Reinforcement (TL, SCT)	Communication from senior staff
Support	SU12.1, SU12.2, SU12.3, SU12.4, SU13.1, SU13.2, SU14.1, SU14.2, SU14.3, SU15.1, SU16.1	Facilitation (SCT)	Communication from senior staff<br>Inclusive language<br>Grief and loss support<br>Scheduled catch ups and check ins<br>Access to support person<br>Access to employee assistance program
Work System	WS13.1, WS13.2	Feedback (TL, SCT)	Regular meetings to provide support<br>Scheduled catch ups and check ins
	SW12.1, WS13.1, WS13.2, WS14.1, WS15.1, WS16.1	Facilitation (SCT)	Access to support person<br>Top-down modelling<br>Scheduled catch ups and check ins
Training and Education	TE12.1, TE12.2, TE12.3, TE13.1, TE13.2, TE13.1, TE14.1, TE14.2, TE14.3, TE15.1, TE16.1	Facilitation (SCT)	Training and education
Environment	ENV12.1, ENV12.2, ENV14.1, ENV15.1	Facilitation (SCT)	Options for debrief<br>Scheduled catch ups and check ins<br>Regular meetings to provide support<br>Communication from senior staff

Abbreviations: HBM – Health Belief Model, TIP – Theories of Information Processing, TTM – Trans-Theoretical Model, SCT – Social Cognitive Theory, ELM – Elaboration Likelihood Model, CPM – Communication Persuasion Matrix, TL – Theories of Learning, TSD – Theories of Stigma and Discrimination, DIT – Diffusion of Innovations Theory

Supplement Table S8

Feasibility Study Findings

Question	Min	Max	Median
Ease of use			
The Portal was easy to use	4	5	5 (strongly agree)
The language used in the Portal was easy to follow	4	5	5 (strongly agree)
I could understand and apply the information given to me in the Portal	4	5	5 (strongly agree)
Using the Portal did not require too much time or effort	4	5	4.5 (agree to strongly agree)
Using the Portal did not inconvenience me	4	5	5 (strongly agree)
I knew how to use and navigate the Portal	3	5	4.5 (agree to strongly agree)
I had the necessary skills to use the Portal	4	5	4.5 (agree to strongly agree)
I was able to access the Portal whenever I wished	4	5	5 (strongly agree)
The design (aesthetic) of the Portal was appealing	3	5	4 (agree)
Usefulness to self and others			
The Portal was useful	4	5	4.5 (agree to strongly agree)
I am satisfied with the Portal	3	5	4 (agree)
The Portal aligned with my personal values	4	5	4 (agree)
I had adequate support and contact with researchers while using the Portal	3	5	4 (agree)
The Portal would be of interest to employees who are in the preconception, pregnancy, or postpartum life stages	4	5	5 (strongly agree)
The Portal would encourage greater consistency of support and understanding from management and colleagues in relation to wellbeing, PPP (preconception, pregnancy, or postpartum), or family needs	4	5	5 (strongly agree)
The Portal would help foster open communication and a supportive culture around HiPPP and family needs at MacKillop	4	5	5 (strongly agree)
The Portal could help me connect with others in a similar life stage at MacKillop	1	4	3 (neither agree or disagree)
I would recommend the Portal to other employees at Mackillop	3	5	5 (strongly agree)
Accessing information through the Portal was better than the MacKillop alternative (i.e., the intranet)	3	5	4.5 (agree to strongly agree)
The Portal could help MacKillop employees keep in touch with those on parental leave and support their transition back to work	3	5	3.5 (neither agree or disagree to agree)
I do not perceive any risks to using the Portal	4	5	4 (agree)
Goals and objectives of the Portal			
The Portal helped increase my knowledge of supports available to me at MacKillop to support my family and HiPPP needs	4	5	5 (strongly agree)
The Portal helped increase my confidence that my HiPPP needs would be met and understood	3	5	4.5 (agree to strongly agree)
The Portal helped me develop my capacity and skills to engage with supports	3	5	4.5 (agree to strongly agree)
The Portal helped me understand my HiPPP rights and responsibilities	4	5	4.5 (agree to strongly agree)

The Portal helped me develop the skills and knowledge to manage my HiPPP, caring and health needs with work	3	5	4.5 (agree to strongly agree)
The Portal helped me develop my skills and knowledge to manage changes to working conditions brought about by COVID	2	5	3.5 (neither agree or disagree to agree)
The Portal helped me connect with those in a similar life stage at MacKillop	2	5	3 (neither agree or disagree)
The components and content of the Portal reflected the intended aim of the study, i.e., to support and understand the health and wellbeing needs of all MacKillop employees and encourage their safety, comfort, knowledge, and belonging during all stages of HiPPP and parenting	3	5	4.5 (agree to strongly agree)
